# Musculoskeletal Pain, Insomnia and Health‐Related Quality of Life: Associations in the Middle‐Aged General Population

**DOI:** 10.1002/ejp.70197

**Published:** 2026-01-05

**Authors:** Tuukka Korpela, Jaro Karppinen, Petteri Oura, Markus Paananen, Arnold Y. L. Wong, Ilona Merikanto, Eveliina Heikkala

**Affiliations:** ^1^ University of Oulu Oulu Finland; ^2^ Research Unit of Health Sciences and Technology, Faculty of Medicine University of Oulu Oulu Finland; ^3^ Medical Research Center Oulu Oulu University Hospital and University of Oulu Oulu Finland; ^4^ Wellbeing Services County of North Ostrobothnia Oulu Finland; ^5^ Wellbeing Services County of South Karelia Lappeenranta Finland; ^6^ Research Unit of Population Health University of Oulu Oulu Finland; ^7^ Western Uusimaa Wellbeing Services County Western Uusimaa Finland; ^8^ Department of Rehabilitation Sciences The Hong Kong Polytechnic University Hong Kong SAR China; ^9^ Research Institute for Smart Ageing The Hong Kong Polytechnic University Hong Kong SAR China; ^10^ SleepWell Research Program, Faculty of Medicine University of Helsinki Helsinki Finland; ^11^ Orton Orthopaedics Hospital Helsinki Finland; ^12^ Wellbeing Services County of Lapland Rovaniemi Finland

**Keywords:** cohort study, health‐related quality of life, insomnia, middle‐aged population, musculoskeletal pain

## Abstract

**Background:**

Musculoskeletal (MSK) pain and insomnia are interrelated and individually linked to reduced health‐related quality of life (HRQoL). This study aimed to enlighten the association of concurrent disabling MSK pain and insomnia with HRQoL among middle‐aged individuals.

**Methods:**

Members of the Northern Finland Birth Cohort 1966 were surveyed at age 46 years (analytic sample *N* = 4 130, 56.7% women). Validated Athens insomnia scale was used to determine insomnia status and HRQoL was assessed with the 15D (15‐dimensional) questionnaire. Associations between different combinations of disabling MSK pain and insomnia (concurrent, isolated, absent) with HRQoL were analysed using general linear regression, adjusting for sex, smoking, educational level, physical activity and coexisting diseases. Fifteen HRQoL dimensions were also analysed separately.

**Results:**

The prevalence of concurrent disabling MSK pain and insomnia was 14.3%, while 50.8% reported neither condition. Adjusted 15D mean score of participants with concurrent disabling MSK pain and insomnia was significantly lower (beta [*β*] coefficient: −0.068 [95% CI −0.073 to −0.062]) compared to those without. Concurrent conditions were associated with lower HRQoL than isolated insomnia and isolated disabling MSK pain (*β* coefficients: −0.032 [95% CI −0.039 to −0.024] and −0.042 [95% CI −0.050 to −0.034], respectively). Fourteen of 15 HRQoL dimensions were the lowest among participants with concurrent conditions.

**Conclusions:**

This study shows that every seventh of the study population has disabling MSK pain and insomnia concurrently and they are more strongly associated with lower HRQoL compared to isolated or absent conditions. The negative effects of comorbid conditions on HRQoL are reflected across multiple dimensions.

**Significance Statement:**

Musculoskeletal pain and insomnia together exert a compounded negative effect on health‐related quality of life, highlighting the need for integrated management strategies. Treating pain alone may not be enough and routine sleep assessment could be a critical point.

## 1 Introduction

Approximately every third person is affected by musculoskeletal (MSK) problems, which cause enormous burden worldwide (Cimmino et al. 2011). Especially in developed countries, MSK problems are among the top 10 reasons for primary healthcare visits (Finley et al. 2018), and MSK pain, the primary symptom of these conditions, incurs substantial costs due to treatments, healthcare usage and work disability (Gorasso et al. 2023). Importantly, MSK pain is known to be associated with poorer health‐related quality of life (HRQoL) (Andersen et al. 2014).

Insomnia is a significant health issue affecting about one‐third of adults (Morin and Jarrin 2022). In middle age, nighttime awakenings, wakefulness after sleep onset, early morning awakenings and shorter total sleep duration tend to become increasingly common compared to younger populations (Boyle et al. 2022). These disturbances with sleep closely resemble the symptoms of insomnia, which is characterised by difficulties initiating or maintaining sleep or non‐restorative sleep occurring at least three times a week and lasting for a minimum of 1 month (World Health Organization 1992). Insomnia is associated with impaired daytime functioning, fatigue, mood disturbances and lower cognitive performance, and most importantly with a markedly lowered HRQoL (Dai et al. 2019). While treating insomnia could improve HRQoL, it remains unclear whether these improvements are clinically meaningful (Kyle et al. 2010). Therefore, further identifying subgroups that are most vulnerable to compromised HRQoL is important.

MSK pain and insomnia are strongly correlated, with a reciprocal relationship (Husak and Bair 2020). People with concurrent chronic pain and insomnia report more intense pain, higher pain‐related disability levels and more severe insomnia than those with only one condition (Dragioti et al. 2018; Husak and Bair 2020). Additionally, healthcare costs appear to be higher for those with co‐occurring chronic pain and insomnia (Dragioti et al. 2018). Middle‐aged and older adults with low back pain or chronic pain often experience more severe insomnia and lower HRQoL compared to age‐matched controls (Abbasi et al. 2018; Dragioti et al. 2018). These findings highlight the importance of addressing MSK pain and insomnia concurrently in terms of HRQoL. To date, a comprehensive comparison of all possible combinations of concurrent MSK pain and insomnia in relation to HRQoL is lacking and thus additional research is warranted to clarify the associations.

The aim of this study is to examine whether concurrent disabling MSK pain and insomnia are associated with HRQoL and to compare this association with isolated disabling MSK pain, isolated insomnia and the absence of these conditions in the general middle‐aged population. We hypothesised that concurrent disabling MSK pain and insomnia would have the strongest association with HRQoL compared to other combinations.

## 2 Methods

### 2.1 Study Population

**FIGURE 1:**
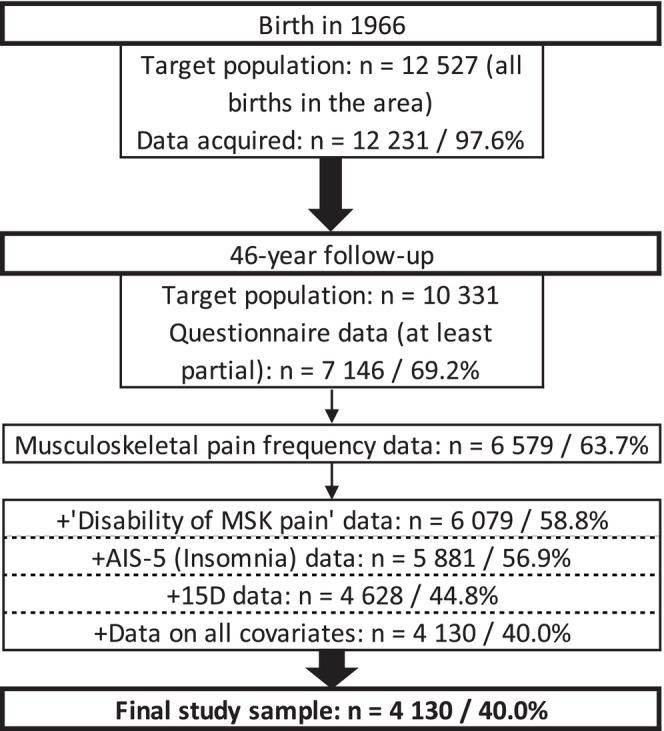
Flowchart of data collection of study sample. 46‐year follow‐up was performed between 2012 and 2014. MSK = musculoskeletal, 15D is a 15‐dimensional health‐related quality of life questionnaire.

The study population consists of individuals in the Northern Finland Birth Cohort 1966 (NFBC1966). The NFBC1966 was formulated from pregnant women who lived in the Northern Finnish provinces of Oulu and Lapland, with expected delivery dates between 1 January and 31 December 1966. The children of NFBC1966 (*n* = 12 527) (hereafter participants) have been followed regularly by questionnaires and clinical examinations. This study used cross‐sectional data collected from participants at the age of 46 (data collection was performed between 2012 and 2014). Data was collected by four questionnaires sent to the participants who were alive and whose addresses were known (*n* = 10 331). Complete information was available from 4130 participants, who provided their written consent for research purposes, constructing the final study sample (Figure 1) (University of Oulu 1966). The study was performed according to the Declaration of Helsinki and it was approved by the Northern Ostrobothnia Hospital District Ethical Committee 94/2011 (12.12.2011).

### 2.2 Musculoskeletal Pain

MSK pain status was dichotomized as ‘disabling MSK pain' and ‘no disabling MSK pain’ using questionnaire‐based information on pain frequency and disability.

#### 2.2.1 Pain Frequency

Participants’ MSK pain frequency was inquired by a question: ‘Have you had pain or ache in the following body parts within the last 12 months? (1) neck or back of head (local or radicular), (2) shoulder, (3) arms or elbows, (4) wrists, hands, or fingers, (5) low back (local, radicular or over 3 months lasting radicular), (6) hips, (7) knees and (8) ankles or feet’. The response options were (1) no, (2) on 1–7 days, (3) on 8–30 days, (4) on more than 30 days but not daily and (5) daily. For every participant, pain frequency was assessed collectively across all body parts. ‘No prolonged pain’ was defined as pain frequency of no more than ‘8–30 days’ for all locations. To be categorised as ‘prolonged pain’, a participant was required to have had pain in at least one location for over 30 days or daily. Participants with incomplete responses were excluded (Figure 1).

#### 2.2.2 Pain Disability

The MSK pain‐related disability was determined by the question: ‘If you had any MSK pain during last 12 months, how disabling have you experienced it?’. The level of disability was asked separately during work, leisure time and sleep, with a scale from 0 to 10 (0 = no disability, 10 = prevents action). Disability was regarded as significant if a participant scored six or more in at least one of the scales (Boonstra et al. 2014).

#### 2.2.3 Disabling Pain

The above‐presented dimensions were used to evaluate the existence of disabling MSK pain. Participants were classified as having disabling MSK pain if they reported prolonged pain and significant disability. If either criterion was not met, they were not considered as having disabling MSK pain.

### 2.3 Insomnia

Sleep issues were evaluated using the five‐item Athens insomnia scale (AIS‐5), which examines participants’ problems with sleep initiation, nighttime awakenings, early morning awakenings, total sleep duration and overall sleep quality. Items correspond to criterion A, while the requirements for frequency (at least three times a week) and duration (1 month) correspond to criterion B of the ICD‐10 classification for the diagnosis of insomnia (World Health Organization 1992). Each item is rated from 0 to 3, with a total score from 0 to 15. Participants were dichotomized as ‘No insomnia’ (under four points) or ‘Insomnia’ (four or more points) (Enomoto et al. 2018). AIS‐5 is a valid and reliable tool for general and chronic pain populations (Enomoto et al. 2018; Soldatos et al. 2000).

### 2.4 Musculoskeletal Pain and Insomnia Status

Information on MSK pain and insomnia was combined to form four MSK pain and insomnia status groups: (1) concurrent disabling MSK pain and insomnia; (2) isolated insomnia; (3) isolated disabling MSK pain; and (4) no disabling MSK pain nor insomnia.

### 2.5 Health‐Related Quality of Life

We used standardised and validated 15D (15‐dimensional) questionnaire to assess participants’ HRQoL. The 15D assesses 15 dimensions affecting quality of life (mobility, vision, hearing, breathing, sleeping, eating, speech, excretion, usual activities, mental function, discomfort and symptoms, depression, distress, vitality and sexual activity) (Sintonen 2001). Each dimension has five response options (no, slight, considerable, severe, or unbearable problems) and standardised multipliers to calculate the 15D score, fitted from 0 to 1 (0 = dead, 1 = no problems in any dimension). Each dimension can also be assessed separately to compare separate dimensions between participants. In all analyses, 15D was used as a continuous variable. Alanne et al. (2015) defined the minimally important change (MIC) value as 0.015, which is considered the smallest change in 15D score and HRQoL that one can feel (hereafter ‘lower HRQoL’). A score change of 0.035 is associated with much‐changed perceived HRQoL (hereafter ‘much lower HRQoL’).

### 2.6 Confounders

Potential confounding variables were chosen a priori based on previous literature suggesting possible associations with MSK pain, insomnia and HRQoL.

#### 2.6.1 Sex

Sex is known to be associated with MSK pain, sleep quality and HRQoL (Fillingim et al. 2009; Zack and Centers for Disease Control 2013; Zhang and Wing 2006). In the analyses, sex was treated as a dichotomized variable, females and males, based on birth record data.

#### 2.6.2 Smoking

Smoking has numerous adverse effects on health and well‐being (Abbafati et al. 2020). It has also been shown to be associated with MSK pain and sleep (Cimmino et al. 2011; Huang et al. 2024). To evaluate smoking habits, we categorised participants into three groups: (1) current smoker; (2) former smoker; and (3) nonsmoker, based on responses to two questions: ‘Have you ever smoked cigarettes?’ and ‘Do you currently smoke?’

#### 2.6.3 Educational Level

Educational level is known to be separately related to MSK pain (Cimmino et al. 2011), short sleep (Stamatakis et al. 2007) and HRQoL (Kahneman and Deaton 2010). Educational level was inquired using the questions: ‘What is your basic education?’ and ‘What is your vocational education?’ Participants were divided into three groups: (1) compulsory or no education (maximum of 9 years of education); (2) secondary (upper secondary or vocational school, 10–12 years of education); and (3) tertiary (university or university of applied sciences, over 12 years of education).

#### 2.6.4 Physical Activity

Physical activity is associated with MSK pain, sleep quality and HRQoL (Cimmino et al. 2011; Kredlow et al. 2015; Pedersen and Saltin 2015). Participants were classified into four groups of leisure‐time physical activity based on the question: ‘How often do you do brisk (get out of breath or sweat at least mildly) exercise during your free time?’ The groups were: (1) less than once a week; (2) once a week; (3) 2–3 times a week; and (4) at least 4 times a week.

#### 2.6.5 Coexisting Diseases

Chronic diseases often interact with MSK pain, sleep and HRQoL (Hale et al. 2020; Heikkala et al. 2023). Participants were asked if they had various diseases, symptoms and traumas diagnosed by physicians. We separately assessed each chronic disease and obesity (self‐reported body mass index over 30 kg/m^2^) to see if they were independently associated with both (1) MSK pain and insomnia status and (2) HRQoL. Diseases associated with both (listed in the footnote of Table 1) were combined into a ‘Coexisting diseases’ variable, which was dichotomized as (1) having at least one coexisting disease and (2) no coexisting diseases. Participants were included if they answered at least one disease‐related question, with blanks regarded as ‘no disease’ to ensure sufficient statistical power in the analysis as only a minor subset had responded to all the items.

### 2.7 Statistical Analyses

All statistical analyses were performed using IBM SPSS version 26.0.0.1 with a statistical significance level set at 0.05. We used crosstabulation with *χ* ^2^ tests to estimate distributions (percentages and numbers) of categorised variables within MSK pain and insomnia status groups, and to examine distributions of MSK pain frequency and disability level between dichotomized insomnia groups. Means with standard deviations were calculated for continuous variables. The Kruskal–Wallis test was utilised to assess the statistical differences in means of single 15D dimensions between MSK pain and insomnia status groups. Representativeness of the study sample was evaluated by comparing participants to nonparticipants using cross‐tabulations, *χ* ^2^ tests, and Mann–Whitney *U*‐tests.

To explore associations of potential confounders with both the MSK pain and insomnia status and HRQoL, we ran univariate multinomial logistic (odds ratios [ORs] and 95% confidence intervals [CIs]) and general linear regression models (beta [*β*] coefficients and 95% CIs), respectively.

For the main analysis, general linear regression models were utilised to obtain *β* coefficients and their 95% CIs for the associations between MSK pain and insomnia status and HRQoL. ‘No disabling MSK pain nor insomnia’ was the primary reference group in these analyses. To study whether ‘concurrent disabling MSK pain and insomnia’ has the strongest association with HRQoL, we also conducted head‐to‐head comparisons using different groups as the reference (e.g., ‘concurrent disabling MSK pain and insomnia’ vs. ‘isolated insomnia’). Models were run as unadjusted and adjusted for all confounders. General linear regression was used also to test the interaction term disabling MSK pain*Insomnia in relation to HRQoL to study whether the association is additive or synergistic.

## 3 Results

A total of 4 130 participants from the NFBC1966 met the inclusion criteria. Half (50.8%, *n* = 2099) of the study population had no disabling MSK pain nor insomnia (Table 1). Concurrent disabling MSK pain and insomnia was reported by 14.3% (*n* = 592), isolated disabling MSK pain by 13.7% (*n* = 566) and isolated insomnia by 21.1% (*n* = 873). Over half (51.1%, *n* = 592 of 1158) of the participants with disabling MSK pain also had insomnia. Likewise, 40.4% (*n* = 592 of 1465) of those with insomnia had disabling MSK pain. In the whole study population, the prevalence of disabling MSK pain was 28.0% and insomnia was 35.5%. Analysis of representativeness revealed no significant differences between the study sample and nonrespondents in the main variables (Appendix S1). However, minor differences in the distributions of confounders and pain frequency were seen.

**TABLE 1:** Demographics of the Northern Finland birth cohort 1966 at the age of 46 stratified by musculoskeletal (MSK) pain and insomnia status.

	MSK pain and insomnia status				
	Total	No disabling MSK pain nor insomnia	Isolated disabling MSK pain	Isolated insomnia	Concurrent disabling MSK pain and insomnia
	(*n* = 4130)	(50.8%, *n* = 2099)	(13.7%, *n* = 566)	(21.1%, *n* = 873)	(14.3%, *n* = 592)
	% (*n*)	% (*n*)	% (*n*)	% (*n*)	% (*n*)
Sex					
Men	43.3 (1789)	46.0 (966)	36.9 (209)	45.9 (401)	36.0 (213)
Women	56.7 (2341)	54.0 (1133)	63.1 (357)	54.1 (472)	64.0 (379)
Smoking					
Current smokers	23.8 (984)	21.5 (452)	26.9 (152)	24.2 (211)	28.5 (169)
Former smokers	27.5 (1136)	26.4 (555)	28.1 (159)	27.0 (236)	31.4 (186)
Nonsmokers	48.7 (2010)	52.0 (1092)	45.1 (255)	48.8 (426)	40.0 (237)
Education level					
Compulsory or no education	5.3 (217)	4.1 (86)	5.1 (29)	6.1 (53)	8.3 (49)
Secondary	65.6 (2710)	64.0 (1344)	68.9 (390)	66.0 (576)	67.6 (400)
Tertiary	29.1 (1203)	31.9 (669)	26.0 (147)	27.9 (244)	24.2 (143)
Level of physical activity					
Less than once a week	26.2 (1084)	22.3 (469)	28.3 (160)	30.2 (264)	32.3 (191)
Once a week	22.1 (912)	22.2 (465)	24.7 (140)	22.9 (200)	18.1 (107)
2–3 times a week	36.2 (1493)	37.2 (780)	34.8 (197)	35.7 (312)	34.5 (204)
At least 4 times a week	15.5 (641)	18.3 (385)	12.2 (69)	11.1 (97)	15.2 (90)
Any coexisting diseases^a^					
Yes	60.1 (2482)	52.3 (1 098)	66.1 (374)	64.9 (567)	74.8 (443)
No	39.9 (1648)	47.7 (1001)	33.9 (192)	35.1 (306)	25.2 (149)
Health‐related quality of life, 15D score	Mean: 0.927, SD: 0.066	Mean: 0.951, SD: 0.046	Mean: 0.920, SD: 0.062	Mean: 0.910, SD: 0.065	Mean: 0.874, SD: 0.085
MSK pain‐related disability	Mean: 4.24, SD: 2.947	Mean: 2.73, SD: 2.216	Mean: 7.63, SD: 1.256	Mean: 3.28, SD: 2.261	Mean: 7.74, SD: 1.207

Abbreviation: SD, standard deviation.

^a^ Obesity, high blood pressure, diabetes (type I and II), hypo‐ and hyperthyroidism, inflammatory bowel disease, epilepsy, migraine, disorder of the cerebral circulation and other nervous system diseases, mental health problems, addiction, sleep apnea, asthma and COPD.

Preliminary analysis showed that MSK pain frequency and disability level were higher among participants with insomnia compared to those without (Figure S1). However, the mean scores of MSK pain‐related disability were practically the same between participants in the ‘concurrent disabling MSK pain and insomnia’ and ‘isolated disabling MSK pain’ groups (Table 1). Similarly, the mean AIS‐5 scores differed only by 0.61 points (on a 0–15 scale) between the ‘concurrent disabling MSK pain and insomnia’ and ‘isolated insomnia’ groups. Detailed description of the study population is presented in Table 1. A higher percentage of participants with ‘concurrent disabling MSK pain and insomnia’ were women, former or current smokers, had a maximum of secondary education and had at least one coexisting disease. All selected confounders were univariately associated with both MSK pain and insomnia status and HRQoL (Tables S1 and S2).

In the main analysis, both unadjusted and fully adjusted models showed that participants with concurrent disabling MSK pain and insomnia had significantly lower HRQoL compared to those without these conditions, with an adjusted mean difference (*β* coefficient) of −0.068 (95% CI −0.073 to −0.062) (Table 2). Similarly, participants with isolated disabling MSK pain or isolated insomnia also had lower and much lower HRQoL (adjusted *β* coefficient −0.024; 95% CI −0.030 to −0.019; adjusted *β* coefficient − 0.035; 95% CI −0.040 to −0.031, respectively) than the reference group. Head‐to‐head comparisons between MSK pain and insomnia groups (Table S3) showed that ‘concurrent disabling MSK pain and insomnia’ was associated with much lower and lower HRQoL when compared with either ‘isolated disabling MSK pain’ or ‘isolated insomnia’ (adjusted *β* coefficient −0.042, 95% CI −0.050 to −0.034 and adjusted *β* coefficient −0.032, 95% CI −0.039 to −0.024, respectively). In comparison between the ‘isolated disabling MSK pain’ and ‘isolated insomnia’, a significant association was also seen but it did not exceed the MIC (adjusted *β* coefficient −0.011, 95% CI −0.017 to −0.005). Nevertheless, test of interaction term disabling MSK pain*Insomnia on HRQoL did not show statistically significant association (adjusted *β* coefficient −0.008, 95% CI −0.016 to 0.000) (Table S4), suggesting that the found association of conditions with HRQoL is rather additive than synergistic.

**TABLE 2:** Association of musculoskeletal (MSK) pain and insomnia status with health‐related quality of life (HRQoL) at age 46 (*n* = 4130).

MSK pain and insomnia status (*n* = 4 130)	15D *β* (95% confidence interval)
Unadjusted	
Concurrent disabling MSK pain and insomnia (*n* = 592)	**−0.077** (−0.083; −0.072)
Isolated insomnia (*n* = 873)	**−0.041** (−0.046; −0.036)
Isolated disabling MSK pain (*n* = 566)	**−0.031** (−0.036; −0.025)
No disabling MSK pain nor insomnia (*n* = 2099)	Reference
Adjusted^a^	
Concurrent disabling MSK pain and insomnia (*n* = 592)	**−0.068** (−0.073; −0.062)
Isolated insomnia (*n* = 873)	**−0.035** (−0.40; −0.031)
Isolated disabling MSK pain (*n* = 566)	**−0.024** (−0.030; −0.019)
No disabling MSK pain nor insomnia (*n* = 2099)	Reference

*Note:* *β* represents mean difference of HRQoL measured by 15D compared to reference group. Statistically significant values are bolded. Minimally important change in 15D = +/−0.015. Much‐changed HRQoL in 15D = +/−0.035.

^a^ Adjusted for sex, smoking, educational level, level of physical activity and coexisting diseases.

**FIGURE 2:**
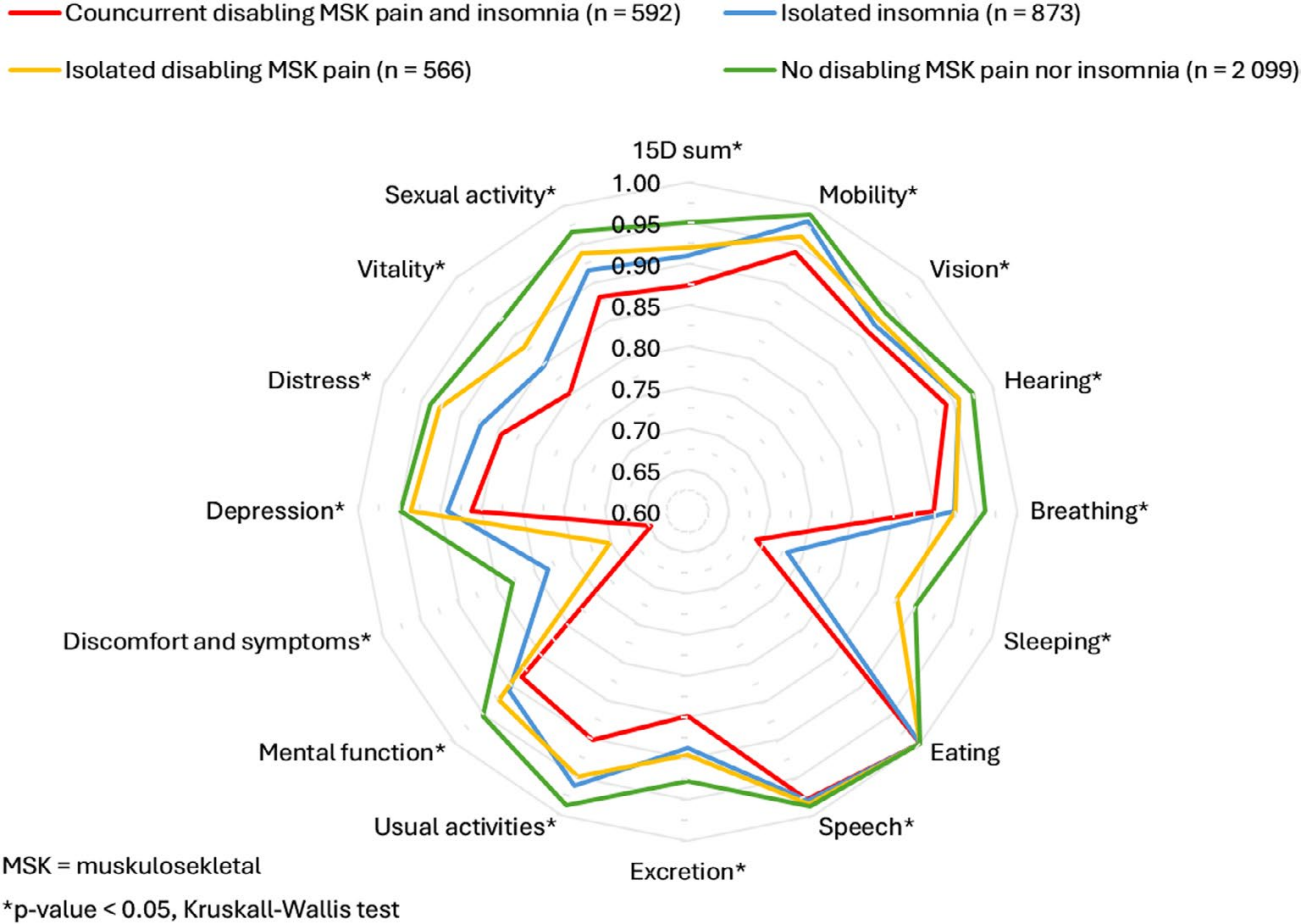
Comparison of single dimension means of 15D among musculoskeletal pain and insomnia status groups.

Figure 2 shows the comparisons of various dimensions of 15D among the four groups. All dimensions except eating differed across four groups (*p* < 0.05), with the greatest differences in single dimensions of ‘sleeping’ and ‘discomfort and symptoms’. Participants with concurrent disabling MSK pain and insomnia scored the lowest mean value for every dimension except eating.

## 4 Discussion

This population‐based study on middle‐aged Northern Finns aimed to determine whether concurrent disabling MSK pain and insomnia are associated with a poorer HRQoL and to compare this association to disabling MSK pain and insomnia in isolation or absence. We found that half of the study population had insomnia or disabling MSK pain, and about one in seven had these conditions concurrently. ‘Concurrent disabling MSK pain and insomnia’ were significantly associated with much lower HRQoL and this finding remained consistent across comparisons with all other MSK pain and insomnia status groups. Similarly, ‘isolated disabling MSK pain’ and ‘isolated insomnia’ were significantly associated with lower HRQoL compared to ‘no disabling MSK pain nor insomnia’, but the strengths of these associations were weaker although clinically meaningful.

The prevalence of co‐occurred disabling MSK pain and insomnia observed in this study was almost identical to a previous estimate from a general population of adults living in Hong Kong (14.6%) (Wong and Fielding 2012). Similarly, the prevalence of disabling MSK pain aligned with the estimates presented in a prior review (13.5%–47%) (Cimmino et al. 2011), as did the prevalence of insomnia with the estimates presented in a review by Morin and Jarrin (2022) (approximately 30%). Over 50% of participants with disabling MSK pain had concomitant insomnia problems and about 40% of those with insomnia also had disabling MSK pain. These observations are also in line with the previous findings showing the prevalence of insomnia symptoms to vary between 41% and 71% among individuals with different pain conditions (Ho et al. 2019). These numbers highlight the prevalence and interconnection of disabling MSK pain and insomnia in the general population, as well as improve the generalizability of our findings.

According to our findings, disabling MSK pain and insomnia, either concurrent or isolated, was associated with a lower HRQoL. These associations remained even after adjusting for confounders. In the adjusted models, both ‘isolated disabling MSK pain’ and ‘isolated insomnia’ were associated with a lower HRQoL when compared to ‘no disabling MSK pain nor insomnia’, with mean difference exceeding the estimated MIC. This was somewhat expected as the literature has shown that MSK pain and insomnia individually tend to contribute to overall well‐being (Kyle et al. 2010; Paananen et al. 2011). Additionally, the strength of these associations was similar to those reported previously for nightly awakenings (Väätäinen et al. 2013) and different MSK pain‐related diseases in relation to HRQoL (Saarni et al. 2006).

Most importantly, we found a stronger association with HRQoL when disabling MSK pain and insomnia coexisted. To our knowledge this is the first study to address these conditions concurrently with HRQoL. Our results showed that concurrent disabling MSK pain and insomnia were associated with much lower HRQoL, with the mean difference against ‘no disabling MSK pain nor insomnia’ being almost twice the much‐changed HRQoL threshold (+/−0.035). Comparisons to ‘isolated disabling MSK pain’ and ‘isolated insomnia’ did not show such great differences, even though they met the MIC and, in the former case, the ‘much lower HRQoL’ threshold. Despite the strong associations of disabling MSK pain and insomnia with HRQoL, a synergistic impact was not found. Instead, a strong additive association was observed, meaning that the impact of these conditions is the sum of their combined effects rather than being amplified by their concurrence. Since there was no significant difference in mean pain‐related disability between the ‘concurrent disabling MSK pain and insomnia’ and ‘isolated disabling MSK pain’, this finding suggests that the strength of the association with much lower HRQoL is not solely attributed to more disabling MSK pain. As the mean AIS‐5 scores between ‘concurrent disabling MSK pain and insomnia’ and ‘isolated insomnia’ differed only slightly, it seems that the severity of insomnia alone does not explain the observed association, unlike what could be expected based on previous literature which found insomnia severity as a significant factor for lower quality of life (Dragioti et al. 2018; Husak and Bair 2020). However, insomnia has shown to be a risk factor for widespread chronic pain, which could be one explanation (Tanguay‐Sabourin et al. 2023). Undoubtedly, further studies are warranted. These results underscore the importance of addressing both disabling MSK pain and insomnia to improve HRQoL and clearly highlight the important role of concurrent conditions in lowered HRQoL. The findings concur with previous results among middle‐aged individuals with low back pain and elderly individuals with chronic pain and associated insomnia experiencing lower quality of life (Abbasi et al. 2018; Dragioti et al. 2018).

Every dimension of HRQoL (except eating) was lower in the concurrent and isolated condition groups compared to the reference group. In line with other findings, the levels were lowest when conditions co‐occurred, indicating that disabling MSK pain and insomnia, particularly when concurrent, is associated with lower HRQoL across all dimensions and not just sleeplessness and pain items, highlighting the broad burden of the phenomenon.

It is known that treatment of even individual MSK pain or insomnia is challenging, and concurrent conditions are even more problematic in terms of sufficient and satisfactory treatment (Ho et al. 2019; Husak and Bair 2020). Cognitive behavioural therapy has been shown to be the most effective non‐pharmacological intervention for improving both insomnia and pain in individuals with chronic MSK pain. However, achieving long‐lasting treatment outcomes and maintaining adherence remain insufficient (Chang et al. 2024). The recent findings regarding treatment methods are largely consistent with earlier studies, suggesting that the results observed in our study population are likely still valid in this regard, despite the passage of over 10 years. The observed strong association between much lower HRQoL and concurrent disabling MSK pain and insomnia might partly explain the difficulties in treatment as lower HRQoL relates also to poorer adherence to treatment (Bernstein et al. 2002). Lower HRQoL has been associated with ‘high cost – high need’ healthcare users (Dragioti et al. 2018; Leininger et al. 2023), higher rates of sick leave among those with pain (Spinord et al. 2022), and lower productivity (Lamers et al. 2005). Thus, avoiding the decrease in HRQoL could potentially lead to immense community savings. However, there is a lack of knowledge on how concurrent MSK pain and insomnia predict work ability (Wong and Fielding 2012). Overall, identifying preventive and predictive factors of concurrent disabling MSK pain and insomnia, as well as exploring their associations with work life, should be a future research priority.

## 5 Strengths and Limitations

We had a large, unselected population‐based cohort that has been followed up regularly. To our knowledge, this is the first population‐based study to evaluate the association of concurrent MSK pain and insomnia with HRQoL. We used a standardised measure with MIC values available for HRQoL, and two important dimensions of MSK pain (frequency and disability) were considered in constructing the MSK pain and insomnia status groups. These support the clinical relevance of the present findings.

Our data were collected via questionnaires and may thus introduce recall bias. However, there are no objective methods available to measure pain in this large scale. Pain is inherently a subjective biopsychological experience, and its objective measurement has also been questioned (Ræder 2017). Our data do not include headaches originating from MSK causes, but only MSK pain in the neck and back of the head. Therefore, the prevalence of MSK‐related headache and its impact on HRQoL may be underestimated. The NFBC1966 is a lifelong cohort study by design, and thus dropouts of participants are inevitable. This has resulted in a minor selection bias towards participants who are women, employed, married and have children (Nordström et al. 2022). However, our study sample still covers approximately 40% of the entire birth cohort. Moreover, we observed no significant differences between our study sample and nonrespondents in terms of main variables, and the observed differences in confounders were minimal, suggesting that our study population adequately represents the entire NFBC1966 population at the 46‐year data collection point. The NFBC1966 does not contain exact data on ethnicity. However, the associations between MSK pain and insomnia have been widely documented across different ethnic groups. There is a mismatch in the assessment periods of insomnia (1 month) and MSK pain (12 months), which could potentially attenuate their interaction term. Nevertheless, as insomnia is measured using the validated AIS‐5 scale aligned with the ICD‐10 diagnostic criteria for insomnia, and MSK pain is assessed as lasting over 30 days, both conditions can be considered as long‐term health disturbances. Therefore, they are likely to be co‐occurring despite the differences in assessment periods. These aspects also highlight the clinical relevance of the variables used, even though they cannot be fully considered as clinical diagnoses due to the epidemiological nature of the study design. Finally, as this study is cross‐sectional, it can only estimate associations and cannot establish cause‐and‐effect relationships.

## 6 Conclusions

The present study based on general population data shows that disabling MSK pain and insomnia are common, with one in seven experiencing both concurrently at the age of 46. We found clear and clinically relevant relationships between disabling MSK pain, insomnia, and lower HRQoL, with the strongest associations observed when both conditions co‐occurred. The reduction was observed across almost every dimension of HRQoL, emphasising the extensive role of these conditions. These results highlight the importance of identifying individuals with both disabling MSK pain and insomnia to develop treatments aiming at maintaining and improving their HRQoL.

## Author Contributions

T.K., J.K., P.O., M.P. and E.H. designed the study setting and planned the implementation of the study. A.Y.L.W. offered insights for study design and offered his expertise about MSK pain. I.M. offered her expertise considering insomnia. T.K. ran all statistical analyses and, together with J.K., P.O., M.P. and E.H., critically examined the results. T.K. had a primary role in preparing the manuscript. All authors edited the manuscript and have revised and approved the final version and agree to be accountable for all aspects of the work.

## Funding

NFBC1966 46y follow‐up study received financial support from University of Oulu Grant no. 24000692, Oulu University Hospital Grant no. 24301140, ERDF European Regional Development Fund Grant no. 539/2010 A31592. This work was supported by Yrjö Jahnsson Foundation under grant number 20227530 and Ane Gyllenberg Foundation under grant number 6414. The funders played no role in the study design, data collection or interpretation, nor in the decision to submit the study for publication.

## Conflicts of Interest

The authors declare no conflicts of interest.

## Supporting information


**Figure S1:** Musculoskeletal (MSK) pain dimensions stratified by insomnia status.

**Table S1:** Confounders‘ associations with concurrency groups.

**Table S2:** Confounders‘ associations with HRQoL. Table shows confounders‘ mean differences of 15D score compared among each confounder. Differences with 95% CIs were obtained through general linear regression models.

**Table S3:** Head‐to‐head comparisons of associations with health‐related quality of life (HRQoL) between different musculoskeletal (MSK) pain and insomnia status groups.

**Table S4:** Association of interaction term of disabling musculoskeletal (MSK) pain*insomnia with health‐related quality of life (HRQoL).

**Appendix S1:** Representativeness of the study sample.

## Data Availability

NFBC data are available from the University of Oulu, Infrastructure for Population Studies. Permission to use the data can be applied for research purposes via an electronic material request portal. In the use of data, we follow the EU general data protection regulation (679/2016) and the Finnish Data Protection Act. The use of personal data is based on a cohort participant's written informed consent in their latest follow‐up study, which may cause limitations to its use. Please contact the NFBC project center (NFBCprojectcenter(at)oulu.fi) and visit the cohort website (www.oulu.fi/nfbc) for more information.

## References

[ejp70197-bib-0001] Abbafati, C. , K. M. Abbas , M. Abbasi‐Kangevari , et al. 2020. “Global Burden of 87 Risk Factors in 204 Countries and Territories, 1990–2019: A Systematic Analysis for the Global Burden of Disease Study 2019.” Lancet 396, no. 10258: 1223–1249. 10.1016/S0140-6736(20)30752-2. 33069327 PMC7566194

[ejp70197-bib-0002] Abbasi, M. , A. M. Kazemifar , H. Fatorechi , and Z. Yazdi . 2018. “Sleep Quality, Quality of Life and Insomnia Among Patients With Chronic Low Back Pain Compared to Normal Individuals.” Sleep and Hypnosis 20, no. 3: 184–189. 10.5350/SLEEP.HYPN.2017.19.0151.

[ejp70197-bib-0003] Alanne, S. , R. P. Roine , P. Räsänen , T. Vainiola , and H. Sintonen . 2015. “Estimating the Minimum Important Change in the 15D Scores.” Quality of Life Research: an International Journal of Quality of Life Aspects of Treatment, Care and Rehabilitation 24, no. 3: 599–606. 10.1007/S11136-014-0787-4. 25145637

[ejp70197-bib-0004] Andersen, L. N. , M. Kohberg , B. Juul‐Kristensen , L. G. Herborg , K. Søgaard , and K. K. Roessler . 2014. “Psychosocial Aspects of Everyday Life With Chronic Musculoskeletal Pain: A Systematic Review.” Scandinavian Journal of Pain 5, no. 2: 131–148. 10.1016/J.SJPAIN.2014.01.001. 29913683

[ejp70197-bib-0005] Bernstein, D. , L. Kleinman , C. M. Barker , D. A. Revicki , and J. Green . 2002. “Relationship of Health‐Related Quality of Life to Treatment Adherence and Sustained Response in Chronic Hepatitis C Patients.” Hepatology 35, no. 3: 704–708. 10.1053/JHEP.2002.31311. 11870387

[ejp70197-bib-0006] Boonstra, A. M. , H. R. S. Preuper , G. A. Balk , and R. E. Stewart . 2014. “Cut‐Off Points for Mild, Moderate, and Severe Pain on the Visual Analogue Scale for Pain in Patients With Chronic Musculoskeletal Pain.” Pain 155, no. 12: 2545–2550. 10.1016/J.PAIN.2014.09.014. 25239073

[ejp70197-bib-0007] Boyle, J. T. , B. Rosenfield , R. A. Di Tomasso , et al. 2022. “Sleep Continuity, Sleep Related Daytime Dysfunction, and Problem Endorsement: Do These Vary Concordantly by Age?” Behavioral Sleep Medicine 21, no. 4: 436–447. 10.1080/15402002.2022.2124994. 36170023 PMC10043048

[ejp70197-bib-0008] Chang, J. R. , Y. K. Cheung , S. Sharma , et al. 2024. “Comparative Effectiveness of Non‐Pharmacological Interventions on Sleep in Individuals With Chronic Musculoskeletal Pain: A Systematic Review With Network Meta‐Analysis.” Sleep Medicine Reviews 73: 101867. 10.1016/j.smrv.2023.101867. 37897843

[ejp70197-bib-0009] Cimmino, M. A. , C. Ferrone , and M. Cutolo . 2011. “Epidemiology of Chronic Musculoskeletal Pain.” Best Practice & Research. Clinical Rheumatology 25, no. 2: 173–183. 10.1016/J.BERH.2010.01.012. 22094194

[ejp70197-bib-0010] Dai, H. , Z. Mei , A. An , and J. Wu . 2019. “Association Between Sleep Problems and Health‐Related Quality of Life in Canadian Adults With Chronic Diseases.” Sleep Medicine 61: 26–30. 10.1016/J.SLEEP.2019.04.015. 31255481

[ejp70197-bib-0011] Dragioti, E. , L. Bernfort , B. Larsson , B. Gerdle , and L. Levin . 2018. “Association of Insomnia Severity With Well‐Being, Quality of Life and Health Care Costs: A Cross‐Sectional Study in Older Adults With Chronic Pain (PainS65+).” European Journal of Pain (London, England) 22, no. 2: 414–425. 10.1002/EJP.1130. 29034538

[ejp70197-bib-0012] Enomoto, K. , T. Adachi , K. Yamada , et al. 2018. “Reliability and Validity of the Athens Insomnia Scale in Chronic Pain Patients.” Journal of Pain Research 11: 793–801. 10.2147/JPR.S154852. 29713192 PMC5907892

[ejp70197-bib-0013] Fillingim, R. B. , C. D. King , M. C. Ribeiro‐Dasilva , B. Rahim‐Williams , and J. L. Riley . 2009. “Sex, Gender, and Pain: A Review of Recent Clinical and Experimental Findings.” Journal of Pain 10, no. 5: 447–485. 10.1016/J.JPAIN.2008.12.001. 19411059 PMC2677686

[ejp70197-bib-0014] Finley, C. R. , D. S. Chan , S. Garrison , et al. 2018. “What Are the Most Common Conditions in Primary Care? Systematic Review.” Canadian Family Physician 64, no. 11: 832. 30429181 PMC6234945

[ejp70197-bib-0015] Gorasso, V. , J. Van der Heyden , R. De Pauw , et al. 2023. “The Health and Economic Burden of Musculoskeletal Disorders in Belgium From 2013 to 2018.” Population Health Metrics 21, no. 1: 4. 10.1186/S12963-023-00303-Z. 37085871 PMC10122398

[ejp70197-bib-0016] Hale, L. , W. Troxel , and D. J. Buysse . 2020. “Sleep Health: An Opportunity for Public Health to Address Health Equity.” Annual Review of Public Health 41: 81–99. 10.1146/ANNUREV-PUBLHEALTH-040119-094412. PMC7944938 31900098

[ejp70197-bib-0017] Heikkala, E. , P. Oura , M. Paananen , et al. 2023. “Chronic Disease Clusters Are Associated With Prolonged, Bothersome, and Multisite Musculoskeletal Pain: A Population‐Based Study on Northern Finns.” Annals of Medicine 55, no. 1: 592–602. 10.1080/07853890.2023.2177723. 36773018 PMC9930817

[ejp70197-bib-0018] Ho, K. K. N. , P. H. Ferreira , M. B. Pinheiro , et al. 2019. “Sleep Interventions for Osteoarthritis and Spinal Pain: A Systematic Review and Meta‐Analysis of Randomized Controlled Trials.” Osteoarthritis and Cartilage 27, no. 2: 196–218. 10.1016/J.JOCA.2018.09.014. 30342087

[ejp70197-bib-0019] Huang, J. , P. Shi , Y. Zhao , H. Zhang , T. Gao , and X. Wang . 2024. “Associations Between Smoking, Sex Steroid Hormones, Trouble Sleeping, and Depression Among U.S. Adults: A Cross‐Sectional Study From NHANES (2013–2016).” BMC Public Health 24, no. 1: 1541. 10.1186/S12889-024-19045-0. 38849814 PMC11157951

[ejp70197-bib-0020] Husak, A. J. , and M. J. Bair . 2020. “Chronic Pain and Sleep Disturbances: A Pragmatic Review of Their Relationships, Comorbidities, and Treatments.” Pain Medicine 21, no. 6: 1142–1152. 10.1093/PM/PNZ343. 31909797

[ejp70197-bib-0021] Kahneman, D. , and A. Deaton . 2010. “High Income Improves Evaluation of Life but Not Emotional Well‐Being.” Proceedings of the National Academy of Sciences of the United States of America 107, no. 38: 16489–16493. 10.1073/PNAS.1011492107. 20823223 PMC2944762

[ejp70197-bib-0022] Kredlow, M. A. , M. C. Capozzoli , B. A. Hearon , A. W. Calkins , and M. W. Otto . 2015. “The Effects of Physical Activity on Sleep: A Meta‐Analytic Review.” Journal of Behavioral Medicine 38, no. 3: 427–449. 10.1007/S10865-015-9617-6. 25596964

[ejp70197-bib-0023] Kyle, S. D. , K. Morgan , and C. A. Espie . 2010. “Insomnia and Health‐Related Quality of Life.” Sleep Medicine Reviews 14, no. 1: 69–82. 10.1016/J.SMRV.2009.07.004. 19962922

[ejp70197-bib-0024] Lamers, L. M. , W. J. Meerding , J. L. Severens , and W. B. F. Brouwer . 2005. “The Relationship Between Productivity and Health‐Related Quality of Life: An Empirical Exploration in Persons With Low Back Pain.” Quality of Life Research: an International Journal of Quality of Life Aspects of Treatment, Care and Rehabilitation 14, no. 3: 805–813. 10.1007/S11136-004-0800-4. 16022073

[ejp70197-bib-0025] Leininger, L. J. , M. Tomaino , and E. Meara . 2023. “Health‐Related Quality of Life in High‐Cost, High‐Need Populations.” American Journal of Managed Care 29, no. 7: 362–368. 10.37765/AJMC.2023.89396. 37523753

[ejp70197-bib-0026] Morin, C. M. , and D. C. Jarrin . 2022. “Epidemiology of Insomnia: Prevalence, Course, Risk Factors, and Public Health Burden.” Sleep Medicine Clinics 17, no. 2: 173–191. 10.1016/J.JSMC.2022.03.003. 35659072

[ejp70197-bib-0027] Nordström, T. , J. Miettunen , J. Auvinen , et al. 2022. “Cohort Profile: 46 Years of Follow‐Up of the Northern Finland Birth Cohort 1966 (NFBC1966).” International Journal of Epidemiology 50, no. 6: 1786–1787J. 10.1093/IJE/DYAB109. 34999878 PMC8743124

[ejp70197-bib-0028] Paananen, M. , S. Taimela , J. Auvinen , T. Tammelin , P. Zitting , and J. Karppinen . 2011. “Impact of Self‐Reported Musculoskeletal Pain on Health‐Related Quality of Life Among Young Adults.” Pain Medicine (Malden, Mass.) 12, no. 1: 9–17. 10.1111/J.1526-4637.2010.01029.X. 21223492

[ejp70197-bib-0029] Pedersen, B. K. , and B. Saltin . 2015. “Exercise as Medicine – Evidence for Prescribing Exercise as Therapy in 26 Different Chronic Diseases.” Scandinavian Journal of Medicine and Science in Sports 25: 1–72. 10.1111/SMS.12581. 26606383

[ejp70197-bib-0030] Ræder, J. 2017. “Objective Measurement of Subjective Pain‐Experience: Real Nociceptive Stimuli Versus Pain Expectation.” Scandinavian Journal of Pain 16: 136–137. 10.1016/J.SJPAIN.2017.05.005. 28850389

[ejp70197-bib-0031] Saarni, S. I. , T. Härkänen , H. Sintonen , et al. 2006. “The Impact of 29 Chronic Conditions on Health‐Related Quality of Life: A General Population Survey in Finland Using 15D and EQ‐5D.” Quality of Life Research: an International Journal of Quality of Life Aspects of Treatment, Care and Rehabilitation 15, no. 8: 1403–1414. 10.1007/S11136-006-0020-1. 16960751

[ejp70197-bib-0032] Sintonen, H. 2001. “The 15D Instrument of Health‐Related Quality of Life: Properties and Applications.” Annals of Medicine 33, no. 5: 328–336. 10.3109/07853890109002086. 11491191

[ejp70197-bib-0033] Soldatos, C. R. , D. G. Dikeos , and T. J. Paparrigopoulos . 2000. “Athens Insomnia Scale: Validation of an Instrument Based on ICD‐10 Criteria.” Journal of Psychosomatic Research 48, no. 6: 555–560. 10.1016/S0022-3999(00)00095-7. 11033374

[ejp70197-bib-0034] Spinord, L. , A. C. Kassberg , B. M. Stålnacke , and G. Stenberg . 2022. “Multivariate Correlations Between Pain, Life Interference, Health‐Related Quality of Life and Full‐Time Sick Leave 1 Year After Multimodal Rehabilitation, Focus on Gender and Age.” Scandinavian Journal of Occupational Therapy 29, no. 8: 645–659. 10.1080/11038128.2021.1903990. 33784480

[ejp70197-bib-0035] Stamatakis, K. A. , G. A. Kaplan , and R. E. Roberts . 2007. “Short Sleep Duration Across Income, Education, and Race/Ethnic Groups: Population Prevalence and Growing Disparities During 34 Years of Follow‐Up.” Annals of Epidemiology 17, no. 12: 948–955. 10.1016/J.ANNEPIDEM.2007.07.096. 17855122 PMC2140008

[ejp70197-bib-0036] Tanguay‐Sabourin, C. , M. Fillingim , G. V. Guglietti , et al. 2023. “A Prognostic Risk Score for Development and Spread of Chronic Pain.” Nature Medicine 29, no. 7: 1821–1831. 10.1038/S41591-023-02430-4. PMC10353938 37414898

[ejp70197-bib-0037] University of Oulu . 1966. University of Oulu: Northern Finland Birth Cohort 1966. http://urn.fi/urn:nbn:fi:att:bc1e5408‐980e‐4a62‐b899‐43bec3755243.

[ejp70197-bib-0038] Väätäinen, S. , H. Tuomilehto , J. Saramies , et al. 2013. “The Health‐Related Quality‐of‐Life Impact of Nocturnal Awakenings in the Middle‐Aged and Older Finnish Population.” Quality of Life Research: an International Journal of Quality of Life Aspects of Treatment, Care and Rehabilitation 22, no. 10: 2737–2748. 10.1007/S11136-013-0404-Y. 23549856

[ejp70197-bib-0039] Wong, W. S. , and R. Fielding . 2012. “The Co‐Morbidity of Chronic Pain, Insomnia, and Fatigue in the General Adult Population of Hong Kong: Prevalence and Associated Factors.” Journal of Psychosomatic Research 73, no. 1: 28–34. 10.1016/J.JPSYCHORES.2012.04.011. 22691556

[ejp70197-bib-0040] World Health Organization . 1992. The ICD‐10 Classification of Mental and Behavioural Disorders: Clinical Descriptions and Diagnostic Guidelines.

[ejp70197-bib-0041] Zack, M. M. , and Centers for Disease Control . 2013. “Health‐Related Quality of Life – United States, 2006 and 2010.” MMWR Supplements 62, no. 3: 105–111. 24264499

[ejp70197-bib-0042] Zhang, B. , and Y. K. Wing . 2006. “Sex Differences in Insomnia: A Meta‐Analysis.” Sleep 29, no. 1: 85–93. 10.1093/SLEEP/29.1.85. 16453985

