# Supplementary material for: Musculoskeletal Pain, Insomnia and Health‐Related Quality of Life: Associations in the Middle‐Aged General Population
Source: Eur J Pain. 2026 Jan 5;30(1):e70197. doi: 10.1002/ejp.70197 (PMC12767138; doi:10.1002/ejp.70197)
Supplement: Supplementary file 1 — Figure S1: Musculoskeletal (MSK) pain dimensions stratified by insomnia status. [file EJP-30-0-s001.docx]

**Figure S1. Musculoskeletal (MSK) pain dimensions stratified by insomnia status.
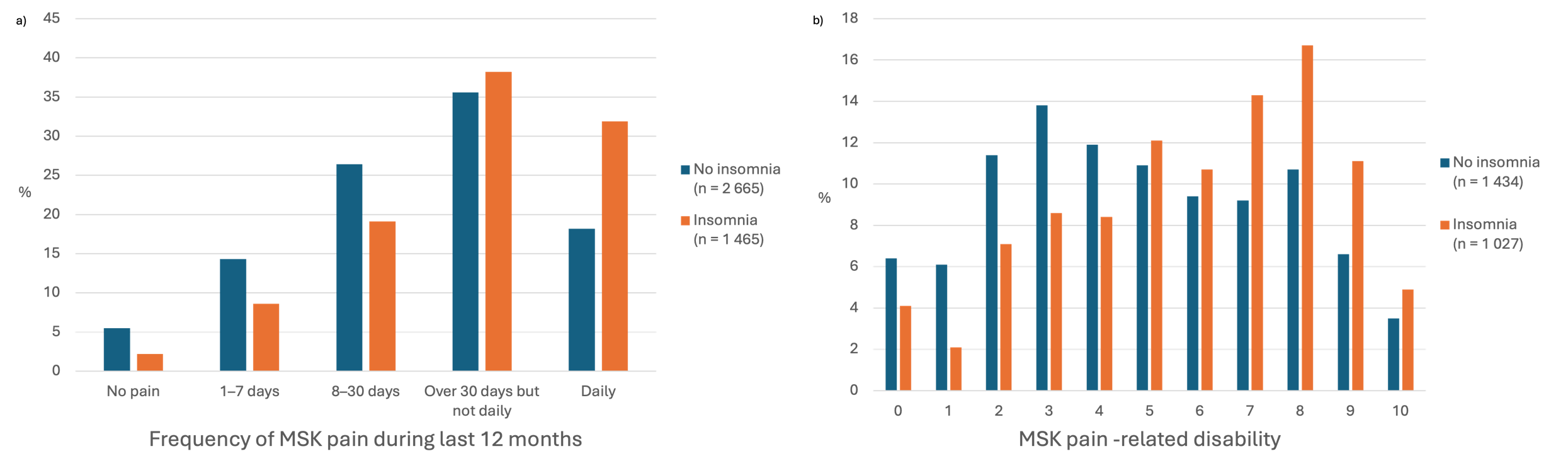
**

1. Pain is reported from eight different locations and the longest duration has been used for determining the frequency. N = 4 130.
2. Disability level is reported on a numerical rating scale 0 to 10 during work, leisure time and sleep. The highest grade of these three dimensions was used. N = 2 461, included only subjects who reported MS pain frequency over 30 days
