# Supplementary material for: Musculoskeletal Pain, Insomnia and Health‐Related Quality of Life: Associations in the Middle‐Aged General Population
Source: Eur J Pain. 2026 Jan 5;30(1):e70197. doi: 10.1002/ejp.70197 (PMC12767138; doi:10.1002/ejp.70197)
Supplement: Supplementary file 2 — Table S1: Confounders‘ associations with concurrency groups. [file EJP-30-0-s003.docx]

## **Table S1. Confounders’ associations with concurrency groups.**

|  | OR (95% CI) | | | | | | | | |  |  |
| --- | --- | --- | --- | --- | --- | --- | --- | --- | --- | --- | --- |
| Confounder | **Sex** | **Smoking** | | **Educational level** | | **Physical activity** | | | **Coexisting diseases** |  |  |
|  |  |  |  |  |  |  |  |  |  |  | |
| Reference group | Men | Non-smoker | | Tertiary | | At least 4 times a week | | | No coexisting diseases |  | |
|  |  |  |  |  |  |  |  |  |  |  | |
| Test group | Women | Former smoker | Current smoker | Secondary | Compulsory or no education | 2-3 times a week | Once a week | Less than once a week | At least one coexisting disease |  | |
|  |  |  |  |  |  |  |  |  |  |  | |
| Concurrent disabling MSK pain and insomnia | **1.517** (1.256; 1.832) | **1.544** (1.242; 1.919) | **1.723** (1.375; 2.159) | **1.392** (1.126; 1.722) | **2.666** (1.797; 3.955) | 1.119 (0.849; 1.475) | 0.984 (0.721; 1.344) | **1.742** (1.311; 2.316) | **2.710** (2.209; 3.325) |  | |
|  |  |  |  |  |  |  |  |  |  |  | |
| Isolated insomnia | 1.004 (0.857; 1.176) | 1.090 (0.902; 1.317) | 1.197 (0.982; 1.459) | 1.175 (0.985; 1.401) | **1.690** (1.164; 2.452) | **1.588** (1.226; 2.056) | **1.707** (1.293; 2.253) | **2.234** (1.707; 2.924) | **1.689** (1.435; 1.090) |  | |
|  |  |  |  |  |  |  |  |  |  |  | |
| Isolated disabling MSK pain | **1.456** (1.203; 1.763) | 1.227 (0.982; 1.533) | **1.440** (1.146; 1.810) | **1.321** (1.069; 1.631) | 1.535 (0.972; 2.424) | **1.409** (1.044; 1.903) | **1.680** ( 1.222; 2.310) | **1.904** (1.392; 2.603) | **1.776** (1.463; 2.156) |  | |
|  |  |  |  |  |  |  |  |  |  |  | |
| No disabling MSK pain nor insomnia | ref. | | | | | | | | |  | |
|  |  |  |  |  |  |  |  |  |  |  | |
| OR = Odds ratio  CI = Confidence interval  Statistically relevant ORs are bolded | | | | | | | | | | |  |
