# Supplementary material for: Musculoskeletal Pain, Insomnia and Health‐Related Quality of Life: Associations in the Middle‐Aged General Population
Source: Eur J Pain. 2026 Jan 5;30(1):e70197. doi: 10.1002/ejp.70197 (PMC12767138; doi:10.1002/ejp.70197)
Supplement: Supplementary file 3 — Table S2: Confounders‘ associations with HRQoL. Table shows confounders‘ mean differences of 15D score compared among each confounder. Differences with 95% CIs were obtained through general linear regression models. [file EJP-30-0-s002.docx]

**Table S2. Confounders’ associations with HRQoL.** Table shows confounders’ mean differences of 15D score compared among each confounder. Differences with 95% CIs were obtained through general linear regression models.

| Confounder | Sex | Smoking | | Educational level | | Physical activity | | | Coexisting diseases |  |
| --- | --- | --- | --- | --- | --- | --- | --- | --- | --- | --- |
|  |  |  |  |  |  |  |  |  |  |  |
| Reference group | Men | Non-smoker | | Tertiary | | At least 4 times a week | | | No coexisting diseases |  |
|  |  |  |  |  |  |  |  |  |  |  |
| Test group | Women | Current smoker | Former smoker | Compulsory or no education | Secondary | Less than once a week | Once a week | 2-3 times a week | At least one coexisting disease |  |
|  |  |  |  |  |  |  |  |  |  |  |
| Mean difference  of 15D (95% CIs) | **-0.011** (-0.015; -0.007) | **-0.022** (-0.027; -0.017) | **-0.009** (-0.014; -0.005) | **-0.033** (-0.042; -0.023) | **-0.010** (-0.015; -0.006) | **-0.037** (-0.044; -0.031) | **-0.016** (-0.023; -0.010) | **-0.010** (-0.016; -0.004) | **-0.034** (-0.038; -0.030) |  |
|  |  |  |  |  |  |  |  |  |  |  |
| CI = confidence interval  Statistically relevant values are bolded | | | | | | | | | |  |
