# Supplementary material for: Musculoskeletal Pain, Insomnia and Health‐Related Quality of Life: Associations in the Middle‐Aged General Population
Source: Eur J Pain. 2026 Jan 5;30(1):e70197. doi: 10.1002/ejp.70197 (PMC12767138; doi:10.1002/ejp.70197)
Supplement: Supplementary file 4 — Table S3: Head‐to‐head comparisons of associations with health‐related quality of life (HRQoL) between different musculoskeletal (MSK) pain and insomnia status groups. [file EJP-30-0-s004.docx]

## **Table S3. Head-to-head comparisons of associations with health-related quality of life (HRQoL) between different musculoskeletal (MSK) pain and insomnia status groups.**

| Concurrent disabling MSK pain and insomnia compared with isolated insomnia (n = 1 465) | 15D ß (95% confidence interval) |
| --- | --- |
| Unadjusted | |
| Concurrent disabling MSK pain and insomnia  (n = 592) | **-0.036** (-0.044; -0.029) |
| Isolated insomnia (n = 873) | Reference |
| Adjusted ^a^ | |
| Concurrent disabling MSK pain and insomnia  (n = 592) | **-0.032** (-0.039; -0.024) |
| Isolated insomnia (n = 873) | Reference |
|  |  |
| Concurrent disabling MSK pain and insomnia compared with isolated disabling MSK pain (n = 1 158) | 15D ß (95% confidence interval) |
| Unadjusted | |
| Concurrent disabling MSK pain and insomnia  (n = 592) | **-0.046** (-0.055; -0.038) |
| Isolated disabling MSK pain (n = 566) | Reference |
| Adjusted ^a^ | |
| Concurrent disabling MSK pain and insomnia  (n = 592) | **-0.042** (-0.050; -0.034) |
| Isolated disabling MSK pain (n = 566) | Reference |
|  |  |
| Isolated insomnia compared with isolated disabling MSK pain (n = 1 439) | 15D ß (95% confidence interval) |
| Unadjusted | |
| Isolated insomnia (n = 873) | **-0.010** (-0.017; -0.003) |
| Isolated disabling MSK pain (n = 566) | Reference |
| Adjusted ^a^ | |
| Isolated insomnia (n = 873) | **-0.011** (-0.017; -0.005) |
| Isolated disabling MSK pain (n = 566) | Reference |

ß represents mean difference of HRQoL measured by 15D between concurrency variable groups

Statistically significant values are bolded

^a^ = Adjusted for sex, smoking, educational level, level of physical activity and coexisting diseases.

Minimally Important Change in 15D = +/-0.015

Much-changed HRQoL in 15D = +/-0.035
