# Supplementary material for: Musculoskeletal Pain, Insomnia and Health‐Related Quality of Life: Associations in the Middle‐Aged General Population
Source: Eur J Pain. 2026 Jan 5;30(1):e70197. doi: 10.1002/ejp.70197 (PMC12767138; doi:10.1002/ejp.70197)
Supplement: Supplementary file 5 — Table S4: Association of interaction term of disabling musculoskeletal (MSK) pain*insomnia with health‐related quality of life (HRQoL). [file EJP-30-0-s005.docx]

**Table S4. Association of interaction term of disabling musculoskeletal (MSK) pain*insomnia with health-related quality of life (HRQoL).**

| Interaction terms | ß | 95% CI | p-value |
| --- | --- | --- | --- |
| Unadjusted | | | |
| Disabling MSK pain*Insomnia | -0.005 | -0.014; 0.003 | 0.205 |
| Adjusted^a^ | | | |
| Disabling MSK pain*Insomnia | -0.008 | -0.016; 0.000 | 0.054 |
| ß represents mean difference of HRQoL measured by 15D ^a^Adjusted for sex, smoking, education level, level of physical activity, coexisting diseases  CI = confidence interval  Reference term for the analyse: No disabling MSK pain*No insomnia | | | |
