# Supplementary material for: Musculoskeletal Pain, Insomnia and Health‐Related Quality of Life: Associations in the Middle‐Aged General Population
Source: Eur J Pain. 2026 Jan 5;30(1):e70197. doi: 10.1002/ejp.70197 (PMC12767138; doi:10.1002/ejp.70197)
Supplement: Supplementary file 6 — Appendix S1: Representativeness of the study sample. [file EJP-30-0-s006.docx]

## **Appendix S1. Representativeness of the study sample (n = 4 130).**

| Variables | Respondents % (n) | Non-Respondents % (n) | p value |
| --- | --- | --- | --- |
| **Sex**^a^ |  |  | < 0.001 |
| Men | 43 (1 789) | 50 (1 497) |  |
| Women | 57 (2 341) | 50 (1 473) |  |
| **MSK pain and insomnia status groups**^a^ |  |  | 0.296 |
| Concurrent disabling MSK pain and insomnia | 14 (592) | 14 (243) |  |
| Isolated insomnia | 21 (873) | 23 (398) |  |
| Isolated disabling MSK pain | 14 (566) | 15 (258) |  |
| No disabling MSK pain nor insomnia | 51 (2 099) | 49 (852) |  |
| **Smoking**^a^ |  |  | < 0.001 |
| current smoker | 24 (984) | 34 (761) |  |
| former smoker | 28 (1 136) | 26 (569) |  |
| non-smoker | 49 (2 010) | 40 (880) |  |
| **Education**^a^ |  |  | < 0.001 |
| compulsory or no education | 5 (217) | 9 (225) |  |
| secondary | 66 (2 710) | 66 (1 578) |  |
| tertiary | 29 (1 203) | 25 (586) |  |
| **Physical activity**^a^ |  |  | 0.001 |
| less than once a week | 26 (1 084) | 30 (769) |  |
| once a week | 22 (912) | 22 (543) |  |
| 2-3 times a week | 36 (1 493) | 32 (813) |  |
| at least 4 times a week | 16 (641) | 16 (404) |  |
| **Coexsisting diseases**^a^ |  |  | 0.009 |
| Yes | 60 (2 482) | 63 (1 666) |  |
| No | 40 (1 648) | 37 (968) |  |
| **15D score**^b^**, mean (SD)** | 0.927 (0.066) | 0.928 (0.066) | 0.274 |
| ^a^ χ^2^-test  ^b^Mann-Whitney U-test  SD = standard deviation  N varies between variable analyses due to missing data  MSK = musculoskeletal | | | |
